# Natural *Leishmania* (*Viannia*) infections of phlebotomines (Diptera: Psychodidae) indicate classical and alternative transmission cycles of American cutaneous leishmaniasis in the Guiana Shield, Brazil

**DOI:** 10.1051/parasite/2017016

**Published:** 2017-05-15

**Authors:** Adelson Alcimar Almeida de Souza, Iorlando da Rocha Barata, Maria das Graças Soares Silva, José Aprígio Nunes Lima, Yara Lúcia Lins Jennings, Edna Aoba Yassui Ishikawa, Ghislaine Prévot, Marine Ginouves, Fernando Tobias Silveira, Jeffrey Shaw, Thiago Vasconcelos dos Santos

**Affiliations:** 1 Seção de Parasitologia, Instituto Evandro Chagas (Secretaria de Vigilância em Saúde, Ministério da Saúde) Ananindeua zip code 67.030-000 Pará State Brazil; 2 Núcleo de Medicina Tropical, Universidade Federal do Pará Belém zip code 66055-240 Pará State Brazil; 3 Département de Médecine, Ecosystèmes Amazoniens et Pathologie Tropicale, EA 3593, Labex CEBA, Université de Guyane zip code 97300 Cayenne French Guiana; 4 Instituto de Ciências Biomédicas, Universidade de São Paulo zip code 05508-000 São Paulo Brazil

**Keywords:** Phlebotomine, Natural infection, *Leishmania*, Guiana Shield

## Abstract

From 1996 to 1999 multi-trapping methods (Center of Diseases Control, CDC) light traps, light-baited Shannon traps, and aspiration on tree bases) were used to study the phlebotomine fauna of the “Serra do Navio” region of the Brazilian State of Amapá, which is part of the Guiana Shield. Fifty-three species were identified among 8,685 captured individuals. The following species, associated with the transmission of American cutaneous leishmaniasis in Amazonian Brazil, were captured: *Nyssomyia umbratilis* (3,388)*, Psychodopygus squamiventris maripaensis* (995)*, Ny. anduzei* (550)*, Trichophoromyia ubiquitalis* (400)*, Ny. whitmani* (291), *Ps. paraensis* (116), and *Bichromomyia flaviscutellata* (50). Flagellate infections were detected in 45 flies. Of the 19 parasites isolated *in vitro,* 15 were *Leishmania* (*Viannia*) *guyanensis* (13 in *Ny. umbratilis*, 1 in *Ny. whitmani*, 1 in *Ny. anduzei*) and three were *L.* (*V.*) *naiffi* (2 in *Ps. s. maripaensis,* 1 in *Ny. anduzei*)*.* The results indicate the participation of three phlebotomine species in the transmission of *L.* (*V.*) *guyanensis* and two species in that of *L.* (*V.*) *naiffi,* and show that the same phlebotomine species is involved in the transmission of different *Leishmania (Viannia)* species in the Guianan/Amazon region. A review of the literature together with the results of the present study, and other published and unpublished results, indicate that eight phlebotomine species potentially participate in the transmission of *Leishmania* (*Viannia*) *naiffi* in Amazonia.

## Abstract (fr)

De 1996 à 1999 plusieurs méthodes de piégeage (pièges lumineux CDC, pièges Shannon et aspirations à la base des arbres) ont été utilisées afin d’étudier les populations de phlébotomes de la région “Serra do Navio” dans l’état brésilien de l’Amapà, qui fait partie du plateau des Guyanes. Cinquante-trois espèces ont été identifiées parmi les 8,685 individus capturés. Plusieurs espèces associées à la transmission de la leishmaniose cutanée américaine en Amazonie brésilienne ont été capturées : *Nyssomyia umbratilis* (3,388)*, Psychodopygus squamiventris maripaensis* (995)*, Ny. anduzei* (550)*, Trichophoromyia ubiquitalis* (400)*, Ny. whitmani* (291), *Ps. paraensis* (116) et *Bichromomyia flaviscutellata* (50). L’infection par des flagellés a été détectée chez quarante-cinq individus. Parmi les 19 isolés *in vitro*, 15 étaient *Leishmania* (*Viannia*) *guyanensis* (13 chez *Ny. umbratilis*, 1 chez *Ny. whitmani*, 1 chez *Ny. anduzei*) et trois étaient *L.* (*V.*) *naiffi* (2 chez *Ps. s. maripaensis,* 1 chez *Ny. anduzei*)*.* Ces résultats indiquent l’implication de trois espèces de phlébotomes dans la transmission de *L.* (*V.*) *guyanensis* et de deux espèces dans la transmission de *L.* (*V.*) *naiffi* et montrent que la même espèce de phlébotome est impliquée dans la transmission de plusieurs espèces de *Leishmania (Viannia)* dans la région guyano-amazonienne. Une revue de la littérature associée aux résultats de la présente étude et à des résultats publiés et non publiés montre que 8 espèces de phlébotomes participent potentiellement à la transmission de *Leishmania* (*Viannia*) *naiffi* en Amazonie.

## Introduction

The Guiana Shield is a geological formation with various ecological areas within the Amazon biome of Venezuela (Delta Amacuro, Bolívar, and Amazonas States), Brazil (Northern Amapá, Pará, Roraima, and Amazonas States), Guyana, Suriname, and the Overseas Department of French Guiana. Its environmental conditions sustain some specific ecological niches and it is one of the regions with the highest biodiversity in the world [49]. Such characteristics favor an array of vector-reservoir relationships and consequently a mosaic of leishmanian ecosystems [55].

American cutaneous leishmaniasis (ACL) is endemic in the region and so far, five dermotropic coexisting *Leishmania* species have been found there: *Leishmania* (*Viannia*) *guyanensis* Floch 1954*, L.* (*V.*) *braziliensis* Vianna 1911*, L.* (*Leishmania*) *amazonensis* Lainson and Shaw 1972*, L.* (*V.*) *lainsoni* Silveira et al. 1987, and *L.* (*V.*) *naiffi* Lainson and Shaw 1989 [55]. *L.* (*V.*) *guyanensis* is the most frequent, accounting for over 80% of ACL cases. However, in French Guiana, for example, recently [43] other species have been found associated with an emerging ACL pattern [*L. (V.) lainsoni –* 1.4%, *L.* (*L.*) *amazonensis* – 2.6%, *L.* (*V*.) *braziliensis* – 8.8%]. These findings are of concern as they indicate ecological changes that favor the transmission of other parasites associated with debilitating forms of the disease, such as diffuse and mucocutaneous leishmaniasis.

Despite the high incidence of ACL in Amapá State (AP) [64], there is a paucity of ecological studies on its vectors and reservoirs. The state is bordered by northern Pará State to the west and French Guiana to the northwest, where the ecology of the disease has been studied more extensively [16, 33, 39, 43, 54, 55]. There are only a few studies on the phlebotomines associated with ACL transmission in AP [5, 15, 17, 18, 46, 58]. The aim of the present survey of the Serra do Navio phlebotomine population is in part to fill this gap by assessing putative transmission cycles in this Brazilian region of the Guiana Shield.

## Materials and methods

### Study area

**Figure 1.**
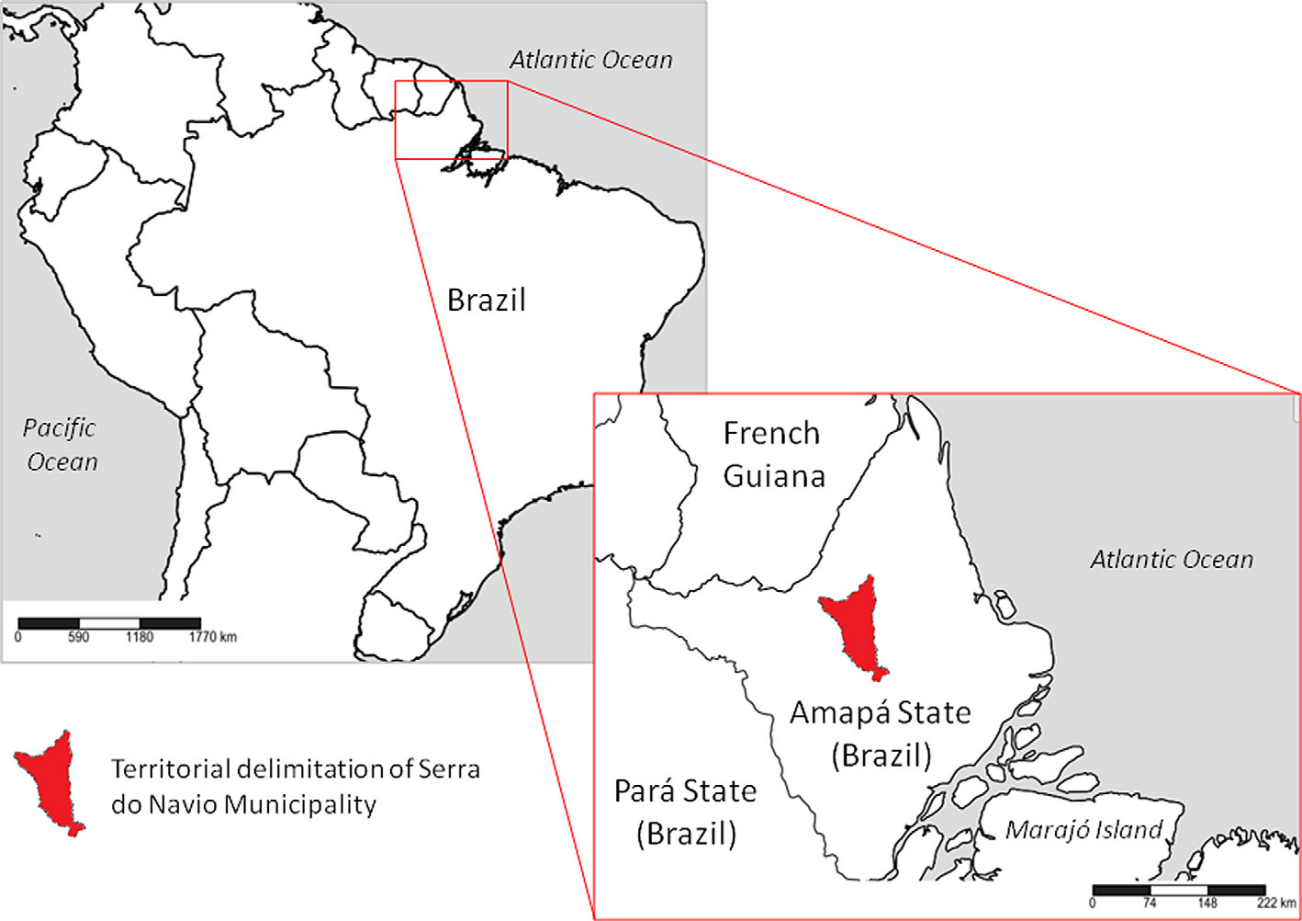
Study area, placed in a Guianan/Amazon forested environment of Serra do Navio municipality, Amapá State, Brazil.

Serra do Navio (00° 53′ 45″  N; 52° 00′ 07″ W, 148 m a.s.l) is one of the 16 municipalities that constitute AP (Northern Brazil). It is in the central area of AP and is 146 km from the state capital, Macapá. Its area is approximately 7,757 km^2^ and it has an estimated population of 4,761 [26] **(** Fig. 1 **)**. The climate is similar to those of the other Amazonian ecoregions of the Guiana Shield, as follows: a short rainy season from mid-November to late January; a short dry season between early February and mid-March; a long rainy season from late March to late July; and a long dry season from late July to mid-November.

Historically, this location is an area of mineral exploitation. In the 1950s the “Sociedade Brasileira de Indústria e Comércio de Minérios de Ferro e Manganês” (ICOMI), in association with Bethlehem Steel, began the manganese mining operation that came to a premature end in 1997. A mining complex was built together with a modern residential township. A health department was set up to look after the health of the mine workers. It was exceptionally efficient and well organized and its team of doctors dealt easily with most of the normal health problems. However, the greatest immediate risks to this isolated population were endemic diseases such as malaria and ACL. The careful use of chloroquinized salt successfully protected them from malaria but no similar measures were or are available against ACL. Today, Serra do Navio is a municipality of intense ACL transmission. During a 2002 epidemiological study, the ACL coefficient of infection was 1,476/100,000 inhabitants [8]. Capture sites were located in three areas of primary forest that were less than 10 km from urban areas. These undisturbed environments were considered to be ecologically similar and were therefore treated as a single site. These areas were predominantly covered by submontane (lowland), dense forest.

### Field and laboratory procedures on phlebotomines

Captures were performed during five 12-night expeditions in 1996 (May and September), 1997 (July and November), and 1999 (October). Traps were installed as follows along transects in forested areas from the edge inwards: eight “Center of Diseases Control” (CDC) light traps set each night at 1.5 m above ground level (6) and at 20 m in the canopy (2), from 06:00 pm to 06:00 am; light-bait Shannon traps, between 06:00 pm and 08:00 pm; and captures on tree bases with a battery-operated aspirator from 07:00 am to 09:00 am. The number of capture hours was calculated by multiplying the number of traps set by the number of hours of exposure. After screening, all phlebotomine females were dissected under sterile conditions as described by Ryan et al. [56]. Males were stored in 70% alcohol. If there was any difficulty in identifying a dissected female, it was mounted in Berlese fluid (GBI Laboratories). Species were identified using Young and Duncan’s guide [74] and the nomenclature adopted is in accordance with the taxonomic criteria proposed by Galati [21]. Two letter genus abbreviations are those suggested by Marcondes [42]. Species composition in the four different trapping samples was analyzed using the Shannon-Wiener diversity index (*H’*) and dominance (*D*), with the aid of the statistical software Past version 2.12 [25].

### Characterization of flagellate strains

The dissected female digestive tracts were examined microscopically for flagellates. Infections were classified according to their distribution in the intestine and semi-quantified using the parasitosis scale adopted by Freitas et al. [17]. After this, the intestine was homogenized and inoculated into two culture tubes containing Difco B^45^ [72]. Isolates were initially characterized by the use of a panel of 23 monoclonal antibodies by a fluorescein-labeled avidin indirect immunofluorescence method (IIF-McAb reaction), as described by Shaw et al. [59]. Phenotypic characterization was undertaken by isoenzyme electrophoresis for _6_PGDH (E.C 1.1.1. 44), PGM (E.C 5.4.2.2), and MPI (E.C 5.3.1.8) as described by Miles et al. [45], and/or by polymerase chain reaction-restriction fragment length polymorphism analysis (PCR-RFLP) of a 615 bp amplified region of the RNA polymerase II gene digested with endonucleases TpsRI and HgaI, as per Simon et al. [63].

McAb, isoenzymatic, and PCR-RFLP profiles of the isolates were compared with those of the five World Health Organization (WHO) reference strains known to occur in the Guiana Shield, *L.* (*L.*) *amazonensis* (IFLA/BR/1967/PH8), *L*. (*V*.) *braziliensis* (MHOM/BR/1975/M2903), *L.* (*V*.) *guyanensis* (MHOM/BR/1975/M4147), *L.* (*V.*) *naiffi* (MDAS/BR/1979/M5533), and *L.* (*V.*) *lainsoni* (MHOM/BR/1981/M6426), as well as with the other WHO reference strains of ecologically closely related species: *L*. (*V*.) *shawi shawi* (MCEB/BR/1984/M8408) and *L.* (*V*.) *lindenbergi* (MHOM/BR/1998/15732).

## Results

The composition of the phlebotomine fauna is summarized in Table 1. A total of 8,685 phlebotomines belonging to 55 taxa were captured by the four trapping methods; of these, 53 were identified to species or subspecies level. Females (6,212) predominated over males (2,473). The species were distributed among 15 genera: *Psathyromyia* (*Pa.,* 10 spp.), *Psychodopygus* (*Ps*., 8 spp), *Evandromyia* (*Ev*., 7 spp.), *Nyssomyia* (*Ny*., 6 spp.), *Micropygomyia* (*Mi*., 3 spp.), *Brumptomyia* (*Br*., 3 spp), *Lutzomyia* (*Lu*., 3 spp*), Pintomyia* (*Pi.,* 3 spp.), *Sciopemyia* (*Sc*., 2 spp), *Trichophoromyia* (*Th*., 2 spp), *Trichopygomyia* (*Ty*., 1 sp.), *Viannamyia* (*Vi*., 2 spp), *Bichromomyia* (*Bi*., 1 spp), *Migonemyia* (*Mg*., 1 sp.), and *Pressatia* (*Pr*., 1 sp). CDC ground captures provided the highest diversity (*H* = 2.627), followed by Shannon traps (2.126), aspiration on tree bases (1.795), and CDC canopy (1.671).

**Table 1.** Species composition, diversity, and infection rate of the Phlebotomine fauna in the Serra do Navio, Amapá State, Brazil, from 1996 to 1999.

*S*	Phlebotomine species	Capture method								Total	%	SIR
		CDC ground		CDC canopy		Shannon		Tree bases				
		♀♀	♂♂	♀♀	♂♂	♀♀	♂♂	♀♀	♂♂			
**1**	***Ny. umbratilis*** (33)	524 (10)	123	1,634 (12)	731	130 (3)	190	16 (8)	40	3,388	38.43	1.43
**2**	***Ps. squamiventris maripaensis*** (2)	156	12	134 (1)	1	627 (1)	63	1	1	995	11.45	0.21
3	*Ev. infraspinosa*	580	36	1	1	5	3			626	7.2	–
4	*Ny. pajoti* (2)	61 (2)	16	230	46	101	161	1	4	620	7.13	0.5
**5**	***Ny. anduzei*** (2)	39 (1)	40	189 (1)	73	26	183			550	6.33	0.78
**6**	***Th. ubiquitalis***	183	138	8	4	31	36			400	4.6	–
**7**	***Ny. whitmani*** (3)	42 (1)	13	168	12	13	2	4 (2)	37	291	3.35	1.32
8	*Ty. trichopyga*	56	95	24	16	7	5			203	2.33	–
9	*Ps. hirsutus*	32	26	48		40	12			158	1.93	–
10	*Vi. tuberculata*	44		94		2		1		141	1.62	–
11	***Ps. paraensis***	24	2	39	2	41	8			116	1.33	–
12	*Ev. bacula*	15		56	2	17	24			114	1.31	–
13	*Vi. furcata*	47		46	1	10	2			106	1.22	–
14	*Lu. gomezi* (1)	38	2	11	2	18 (1)	1	4	27	103	1.18	1.4
15	***Ps. davisi***	23	8	38	5	13	8			95	1.09	–
16	*Pa. aragaoi*	47	20	19	3		2			91	1.04	–
17	*Th. brachipyga*	27	34	3	1	5	2			72	0.82	–
18	*Ps. amazonensis*	16	3	24	4	24				71	0.81	–
19	*Pa. scaffi*		4					8	57	69	0.79	–
20	*Ps. geniculatus*	19	2	14		14	3			52	0.59	–
**21**	***Bi. flaviscutellata***	18	12	7	1	10	2			50	0.57	–
–	*Brumptomyia* spp.	17		29						46	0.52	–
22	*Pa. barretoi barretoi*	1	24		10		1			36	0.41	–
23	*Ps. claustrei*	6	4	6		15				31	0.35	–
24	*Pa. dendrophyla*	14	1	13		1	1		1	31	0.35	–
25	*Ev. monstruosa*	13	1	1		12				27	0.31	–
26	*Ev. evandroi*	15		2		2				19	0.21	–
27	*Mg. migonei* (1)	2 (1)		8	7		2			19	0.21	10
28	*Pi. damascenoi*	8		2		6			2	18	0.20	–
29	*Pa. runoides*	9	2	1	1	1				14	0.16	–
30	*Pa. bigeniculata*	4		1	1			2	6	14	0.16	–
31	*Mi. rorotaensis*	4	1			1		5	3	14	0.16	–
32	*Mi. micropyga*	9		1		1				11	0.12	–
33	*Sc. sordellii* (1)	5	1	1		2 (1)	1			10	0.11	12.5
34	*Ev. sericea*	5	4		1					10	0.11	–
35	*Pa. dreisbachi*			1	8					9	0.10	–
36	*Pr. trispinosa*	1	6	1						8	0.09	–
37	*Lu. carvalhoi*	4		2		1				7	0.08	–
38	*Lu. spatotrichia*	1		3	1		1			6	0.06	–
39	*Ps. carrerai*	2	2	1		1				6	0.06	–
40	*Ny. antunesi*	2	1						2	5	0.05	–
41	*Ev. inpai*	1	3							4	0.04	–
42	*Pa. lutziana*	2		1	1					4	0.04	–
43	*Pa. inflata*	1	2	1						4	0.04	–
44	*Sc. fluviatilis*	2				2				4	0.04	–
45	*Br. travassosi*		2		1					3	0.03	–
46	*Pi. serrana*			2		1				3	0.03	–
47	*Ny. richardwardi*	1		1						2	0.02	–
48	*Pa. abonnenci*		1		1					2	0.02	–
49	*Mi. pilosa*	2								2	0.02	–
50	*Br. beaupertuyi*		2							2	0.02	–
51	*Ev. brachyphalla*	1								1	0.01	–
52	*Pi. pacae*				1					1	0.01	–
53	*Br. pintoi*		1							1	0.01	–
	Total	2,123	644	2,865	938	1,180	713	42	180	8,685		
Diversity	*Taxa* (*S*)	51		45		36		12		54		
	Individuals	2,767		3,803		1,893		222		–		
	Dominance (*D*)	0.1294		0.402		0.1967		0.2064		0.1852		
	Shannon (*H*)	2.627		1.671		2.126		1.795		2.415		

(): Number of individuals found with natural infection by flagellates; **♀♀**: females; **♂♂**: males; bold, species associated with ACL agents occurring in the Guiana Shield on the basis of current literature [6, 16, 53, 55, 65]; SIR: Species Infection Rate.

*Ny. umbratilis* (3,388)*, Ps. s. maripaensis* (995)*, Ny. anduzei* (550)*, Th. ubiquitalis* (400)*, Ny. whitmani* (291), and *Ps. paraensis* (116) were among the ten most frequent species. The dominance (*D* = 0.402) was due to high numbers of *Ny. umbratilis* in canopy CDCs.

The average of phlebotomine putative vector species captured per hour can be found in Table 2. Considering all capture methods, captures of *Ny. umbratilis* (0.64) were the greatest, followed by *Ps. squamiventris maripaensis* (0.19). However, when each trap was compared individually, Shannon captures were higher for *Ps. s. maripaensis* (11.5), *Ny umbratilis* (5.33), and *Ny. anduzei* (3.48).

**Table 2.** Average of phlebotomine putative vector species captured per hour in the Serra do Navio, Amapá State, Brazil, from 1996 to 1999.

Phlebotomine putative vector species	Capture method*				Total**
	CDC ground	CDC canopy	Shannon	Tree bases	
*Ny. umbratilis*	0.17	1.64	5.33	0.93	0.64
*Ps. squamiventris maripaensis*	0.04	0.09	11.5	0.03	0.19
*Ny. anduzei*	0.02	0.18	3.48	–	0.10
*Th. ubiquitalis*	0.08	0.008	1.11	–	0.07
*Ny. whitmani*	0.01	0.12	0.25	0.68	0.05
*Ps. paraensis*	0.007	0.02	0.81	–	0.02
*Bi. flaviscutellata*	0.008	0.005	0.2	–	0.009

* Based on 3,600 h CDC ground, 1,440 h CDC canopy, 60 h Shannon, and 60 h of aspiration on tree bases;

** Based on 5,160 h of total trapping.

There was no evidence of blood in the intestines of the 45 infected females and the parasitosis ranged from 21–40 (+++) to 41 or above (++++) flagellates per field (40× objective). From their morphology, we were unable to confirm that any of these trypanosomatids were *Leishmania*.

Flagellates were found in 0.72% of the females (45/6,212): 33/2,304 *Ny. umbratilis* (12-CDC canopy, 10-CDC ground, 8-Tree bases, 3-Shannon; species infection rate: 1.43); 2/918 *Ps. s. maripaensis* (1-CDC canopy, 1-Shannon; species infection rate: 0.21%); 3/227 *Ny. whitmani* (2-Tree bases, 1-CDC ground; species infection rate: 1.32%); 2/254 *Ny. anduzei* (1-CDC ground, 1-CDC canopy; species infection rate: 0.78%); 2/393 *Ny. pajoti* (CDC ground; species infection rate: 0.5%); 1/10 *Mg. migonei* (CDC ground; species infection rate: 10%); 1/71 *Lu. gomezi* (Shannon; species infection rate: 1.4%); and 1/8 *Sc. sordellii* (Shannon; species infection rate: 12.5%).

**Figure 2.**
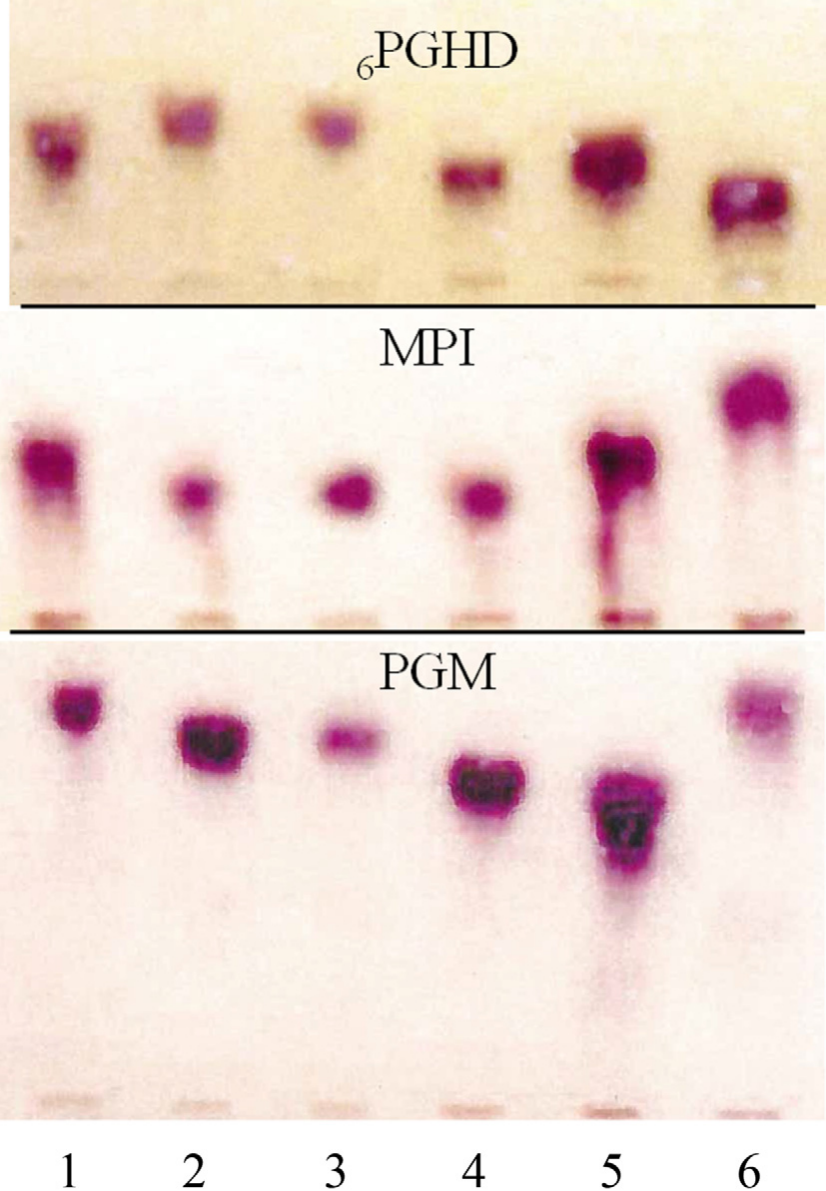
Isoenzyme electrophoresis of 6PGDH, MPI, and PGM enzymatic systems for the isolate from *Nyssomya whitmani* in the “Serra do Navio”, Amapá State, Brazil*,* compared with the WHO reference strains of Brazilian Amazon *Leishmania* species. Reading from left to right: (1) *L. (V.) braziliensis* (MHOM/BR/1975/M2903); (2) IWHI/BR/1997/M16399; (3) *L. (V.) guyanensis* (MHOM/BR/1975/M4147); (4) *L. (V.) s. shawi* (MCEB/BR/1984/M8408); (5) *L.* (*V.*) *naiffi* (MDAS/BR/1979/M5533); (6) *L. (V.) lainsoni* (MHOM/BR/1981/M6426).

**Figure 3.**
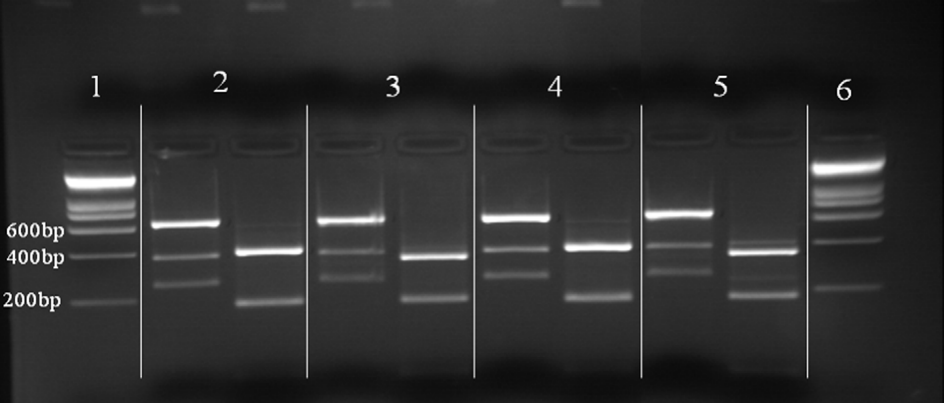
PCR-RFLP analysis of *Leishmania* isolates from *Nyssomyia whitmani* and *Nyssomyia anduzei* in the “Serra do Navio”, Amapá State, Brazil*,* compared with the closely related WHO reference strains of Brazilian Amazon *Leishmania* species, *L. (V.) guyanensis* and *L.* (*V.*) *naiffi*. Reading from left to right: (1) Molecular weight marker Smart Ladder^®^; (2) *L. (V.) guyanensis* (MHOM/BR/1975/M4147); (3) *L.* (*V.*) *naiffi* (MDAS/BR/1979/M5533); (4) IWHI/BR/1997/M16399; (5) IAND/BR/1997/M16408; (6) Molecular weight marker Smart Ladder^®^.

Nineteen flagellate strains were successfully isolated. Fifteen were identified as *L.* (*V.*) *guyanensis* from *Ny. umbratilis* (13), *Ny. whitmani* (1), and *Ny. anduzei* (1), and three as *L.* (*V.*) *naiffi* from *Ps. s. maripaensis* (2) and *Ny. anduzei* (1) (Table 3)*.* Both *Leishmania* species had the same McAb profiles as the WHO reference strains MHOM/BR/1975/M4147 and MDAS/BR/1979/M5533. The *Mg. migonei* isolate (IMIG/BR/1997/M16230) did not react with any of the McAbs. The identifications of the *Ny. anduzei* (IAND/BR/1997/M16408) isolate as *L*. (*V*.) *naiffi* and the *Ny. whitmani* (IWHI/BR/1997/M16399) isolate as *L.* (*V.*) *guyanensis* were confirmed by _6_PGDH/PGM/MPI isoenzyme electrophoresis (Fig. 2) and PCR-RFLP analysis (Fig. 3). Table 4 gives a list of *L. (V.) naiffi* phlebotomine infections prepared from the results of this study and other published records.

**Table 3.** Naturally infected phlebotomine species and their respective results for *Leishmania* spp. isolation/characterization in the Serra do Navio, Amapá State, Brazil, from 1996 to 1999.

N	IEC code	Phlebotomine species	Collection data (trapping/site)	Result*	MCAb reaction-profile	WHO Code
1	M 15928	*Ny. umbratilis*	Shannon	Negative/contaminated	–	–
2	M 15929	*Sc. sordellii*	Shannon	Negative/contaminated	–	–
3	M 15930	*Lu. gomezi*	Shannon	Negative/contaminated	–	–
4	M 15931	*Ny. umbratilis*	CDC canopy	*L.* (*V.*) *guyanensis*	L1; B2; B12V; B19	IUMB/BR/1996/M15931
5	M 15932	*Ny. yuilli pajoti*	CDC ground	Negative/contaminated	–	–
6	M 15933	*Ny. umbratilis*	CDC ground	Negative/contaminated	–	–
7	M 15934	*Ny. umbratilis*	CDC ground	Negative/contaminated	–	–
9	M 15935	*Ny. umbratilis*	CDC ground	Negative/contaminated	–	–
9	M 15936	*Ny. yuilli pajoti*	CDC ground	Negative/contaminated	–	–
10	M 16230	*Mg. migonei*	CDC ground	?	No reaction	IMIG/BR/1997/M16230
11	M 16231	*Ny. umbratilis*	CDC ground	Negative/contaminated	–	–
12	M16232	*Ny. umbratilis*	Shannon	Negative/contaminated	–	–
13	M 16233	*Ny. umbratilis*	CDC canopy	Negative/contaminated	–	–
14	M 16234	*Ny. umbratilis*	Shannon	Negative/contaminated	–	–
15	M 16235	*Ps. squamiventris maripaensis*	CDC canopy	*L.* (*V.*) *naiffi*	L1; B12; N2; N3	ISQU/BR/1997/M16235
16	M 16390	*Ny. umbratilis*	CDC canopy	*L.* (*V*.) *guyanensis*	L1; B2; B12V; B19	IUMB/BR/1997/M16390
17	M 16391	*Ny. umbratilis*	CDC canopy	*L.* (*V*). *guyanensis*	L1; B2; B12V; B19	IUMB/BR/1997/M16391
18	M 16392	*Ny. umbratilis*	CDC canopy	Negative/contaminated	–	–
19	M 16393	*Ny. umbratilis*	CDC canopy	Negative/contaminated	–	–
20	M 16394	*Ny. umbratilis*	CDC ground	Negative/contaminated	–	–
21	M 16395	*Ny. umbratilis*	CDC ground	Negative/contaminated	–	–
22	M 16396	*Ny. umbratilis*	CDC canopy	Negative/contaminated	–	–
23	M 16397	*Ny. umbratilis*	CDC canopy	Negative/contaminated	–	–
24	M 16398	*Ny. umbratilis*	CDC ground	*L. (V*.) *guyanensis*	L1; B2; B12V; B19	IUMB/BR/1997/M16391
25	M 16399	*Ny. whitmani*	CDC ground	*L.* (*V*.) *guyanensis*	L1; B2; B12V; B19	IWHI/BR/1997/M16399
26	M 16400	*Ny. umbratilis*	CDC ground	*L.* (*V*.) *guyanensis*	L1; B2; B12V; B19	IUMB/BR/1997/M16400
27	M 16402	*Ny. umbratilis*	CDC canopy	*L.* (*V*.) *guyanensis*	L1; B2; B12V; B19	IUMB/BR/1997/M16401
28	M 16403	*Ny. anduzei*	CDC ground	*L.* (*V*.) *guyanensis*	L1; B2; B12V; B19	IAND/BR/1997/M16403
29	M 16404	*Ny. umbratilis*	CDC ground	Negative/contaminated	–	–
30	M 16405	*Ny. umbratilis*	CDC ground	*L.* (*V*.) *guyanensis*	L1; B2; B12V; B19	IUMB/BR/1997/M16405
31	M 16406	*Ny. umbratilis*	CDC canopy	Negative/contaminated	–	–
32	M 16407	*Ps. s. maripaensis*	Shannon	*L.* (*V.*) *naiffi*	L1; B12; N2; N3	ISQU/BR/1997/M16407
33	M 16408	*Ny. anduzei*	CDC canopy	*L.* (*V*.) *naiffi*	L1; B12; N2; N3	IAND/BR/1997/M16408
34	M 16409	*Ny. umbratilis*	CDC canopy	*L.* (*V*.) *guyanensis*	L1; B2; B12V; B19	IUMB/BR/1997M16409
35	M 16410	*Ny. umbratilis*	CDC canopy	*L.* (*V*.) *guyanensis*	L1; B2; B12V; B19	IUMB/BR/1997/M16410
36	M 17944	*Ny. whitmani*	Tree bases	Negative/contaminated	–	–
37	M 17945	*Ny. whitmani*	Tree bases	Negative/contaminated	–	–
38	M 17946	*Ny. umbratilis*	Tree bases	*L.* (*V*.) *guyanensis*	L1; B2; B12V; B19	IUMB/BR/1999/M17946
39	M 17947	*Ny. umbratilis*	Tree bases	*L.* (*V.*) *guyanensis*	L1; B2; B12V; B19	IUMB/BR/1999/M17947
40	M 17948	*Ny. umbratilis*	Tree bases	Negative/contaminated	–	–
41	M 17949	*Ny. umbratilis*	Tree bases	Negative/contaminated	–	–
42	M 17950	*Ny. umbratilis*	Tree bases	Negative/contaminated	–	–
43	M 17951	*Ny. umbratilis*	Tree bases	Negative/contaminated	–	–
44	M 17952	*Ny. umbratilis*	Tree bases	*L.* (*V.*) *guyanensis*	L1; B2; B12V; B19	IUMB/BR/1999/M17952
45	M 17953	*Ny. umbratilis*	Tree bases	*L.* (*V.*) *guyanensis*	L1; B2; B12V; B19	IUMB/BR/1999/M17953

* Based on McAb, isoenzyme, and PCR-RFLP profiles.

**Table 4.** Present and literature-based *Leishmania (Viannia) naiffi* detection in phlebotomine species.

Phlebotomine species	Location	*L.* (*V.*) *naiffi* typing	Reference
*Ps. ayrozai* (3)	BR 319, Km 866 (RO), BR	MLEE	Arias et al. (1985) [2]
*Ps. paraensis* (4)	BR 319, Km 866 (RO), BR	MLEE	Arias et al. (1985) [2]
*Ps. squamiventris s.l.* (1)	Balbina (AM), BR	MLEE	Grimaldi et al. (1991) [23]
*Ps. squamiventris s.l.* (1)	Cachoeira Porteira (PA), BR	MLEE	Grimaldi et al. (1991) [23]
*Ps. paraensis* (1)	Benevides (PA)	MLEE	Silveira et al. (1991) [62]
*Ps. ayrozai*	Benevides (PA)	MLEE	Silveira et al. (1991) [62]
*Ps. davisi*	Cacaulândia (RO), BR	McAb	Gil et al. (2003) [22]
*Ps. hirsutus*	Cacaulândia (RO), BR	McAb	Gil et al. (2003) [22]
*Ps. s. maripaensis* (1)	Sinnamary, FG	RNA *poly II* gene sequencing	Fouque et al. (2007) [16]
*Lu. tortura* (1)	Arajuno, EC	*Cyt b* gene sequencing	Kato et al. (2008) [29]
*Ps. davisi* (2)	Belterra (PA), BR	McAb/MLEE	Souza et al. (2016) [65]
*Ps. hirsutus* (1)	Belterra (PA), BR	McAb/MLEE	Souza et al. (2016) [65]
*Ps. wellcomei*/*complexus* (1)	Belterra (PA), BR	McAb/RNA *poly II* gene sequencing	Unpublished
*Ny. anduzei* (1)	Serra do Navio (AP), BR	McAb/RNA *poly II* gene PCR-RFLP	Present study
*Ps. s. maripaensis* (2)	Serra do Navio (AP), BR	McAb/RNA *poly II* gene PCR-RFLP	Present study

*Notes*. All *Leishmania (Viannia) naiffi* detections were based on microscopic analysis of dissected flies and further *in vitro* (direct culture) and/or *in vivo* (inoculation in *hamster* prior culture) parasite isolation. (): number of infected specimens, when available; McAb: Monoclonal antibodies; MLEE: Multilocus enzyme electrophoresis; *poly II*: *polymerase II*; (AM): Amazonas State; (AP): Amapá State; (PA): Pará State; (RO): Rondônia State; BR; Brazil; EC: Ecuador; FG: French Guiana; SU: Suriname.

## Discussion

Amapá State is one of Brazil’s most environmentally preserved regions and the present survey of its phlebotomine fauna amplifies the findings of previous researchers [1, 5, 15, 17, 18, 19, 20, 46, 48, 57, 74]. Five species [*Ev. bacula* (Martins, Falcão & Silva, 1965)*, Pa. runoides* (Farchild & Hertig, 1953)*, Ps. carrerai* (Barretto, 1946)*, Br. beaupertuyi* (Ortiz, 1954) (first record for Brazil), and *Br. pintoi* (Costa Lima, 1932)] were recorded for the first time in this state by this study. Thus, Aguiar and Medeiros’ [1] checklist, which records 55 species for AP, needs updating.

The first records for phlebotomines in the Serra do Navio were produced in the 1960s from collections by JE Silva (unpublished). Young and Duncan [74] and Ward and Fraiha [73] referred to collections made by D. Young in the 1970s, but there are no checklists for this municipality. The specimens collected by JE Silva are deposited in René Rachou’s phlebotomine collection (FIOCRUZ, Belo Horizonte, Minas Gerais, Brazil) and some of them have been used for the description of *Micropygomyia pusilla* (Dias, Martins Falcão & Silva, 1986) [11]. This species, however, was not found in the present survey.

The highest richness of species was observed for *Psathyromyia* and *Psychodopygus,* among the 15 genera captured in Serra do Navio. The former also included four species from the *Shannoni* series Fairchild, 1955, whose taxonomic status has recently been revised [57]. Voucher specimens originally identified as *Pa. shannoni* (Dyar, 1929) (MGS Silva det.) were re-examined and after further consultation (AJ Andrade, personal communication), it was concluded that they in fact represent the resurrected *Pa. bigeniculata* (Floch & Abonnenc, 1941).

The role of different phlebotomine species in ACL epidemiology in the Guiana Shield contrasts with that of the Amazonian lowlands. In the latter region, *Psychodopygus* species appear to be the dominant vectors, whereas in the Guiana Shield, it is the *Nyssomyia* species that dominate.

As might be expected, the principal vector of *L.* (*V.*) *guyanensis*, *Ny*. *umbratilis,* stands out in the list of vectors, in accordance with several other studies in highly endemic ACL regions [33, 50, 51]. A part of the life cycle of *Ny. umbratilis* is arboreal, and this is highlighted by its dominant presence in our canopy traps. In an area affected by a hydroelectric dam project on the Jari River (AP), no differences were observed between ground level and canopy catches for this species. This could be because most arboreal animals, which are the principal blood source for this species, were removed during environmental management operations related to the destruction of the forest [18]. Unfortunately, present captures are logistically biased which often weakens conclusions on seasonality. However, we found that *Ny. umbratilis,* including infected individuals, was only taken from tree trunks at ground level during the dry season. Our data on the tree-dwelling behavior of *Ny umbratilis* are compatible with those on the well-studied populations of the Northern Amazon [39, 54, 73]. The morphologically closely related species found in smaller numbers, *Ny. anduzei,* behaves similarly and it has been suggested that it is a secondary *L.* (*V.*) *guyanensis* vector in ACL foci where *Ny. umbratilis* is present [53].

*Ps. s. maripaensis* has been associated with *L.* (*V.*) *naiffi* in northern Brazil [47] and French Guiana [16], and has been suggested as a possible vector of *L.* (*V.*) *braziliensis* and *L.* (*V*.) *naiffi* in Suriname [30]. Its high numbers in our Shannon captures suggest its anthropophilic behavior and preference for populations living at ground level. Catchers in the Shannon traps constantly found this species trying to bite them despite the use of protective measures. Two infections in *Ps. s. maripaensis* (Shannon and CDC canopy) were characterized as *L*. (*V.*) *naiffi*. Human infections with this parasite are unknown in AP, but these findings are proof that its enzootic is present in the Serra do Navio forest. Records also link its known distribution to this *Leishmania* species in the Brazilian states of Amazonas [23, 47], Acre [61], Rondônia [2, 22], Pará [35] and the belt that includes AP, French Guiana [55], and Suriname [69]. Interestingly, single records for *L.* (*V.*) *naiffi* have also been reported in Ecuador [29], Peru, Martinique (inconclusive?) [52], and Panama [3].

*Leishmania* DNA has been found in some *Trichophoromyia* species and their possible importance in ACL epidemiology has been discussed [30, 44, 67]. *Th. ubiquitalis* is the proven vector of *L.* (*V*.) *lainsoni* [53, 62] and its potential importance in ACL epidemiology in AP is supported by the fact that *L.* (*V*.) *lainsoni* has been diagnosed in patients from this state (FT Silveira, personal observations).

In Pará State, *Ny. whitmani* is associated with transmission of *L.* (*V.*) *shawi shawi* [32] and latterly *Lu. gomezi* has been indicated as a putative vector of this same parasite [65]. However, *Ny. whitmani* has also been associated with the *L. (V.*) *guyanensis* enzootic in the Monte Dourado region of the Guiana Shield [33]. In the north-eastern and Atlantic forest region of Brazil, *Ny. whitmani* is an important vector of *L.* (*V*.) *braziliensis* [4, 6, 70]. In these environments, *Ny. whitmani* occurs predominantly in peri-domestic situations. Its sylvatic occurrence in the present study is in accordance with early studies [35, 60], and may represent a typical Amazonian population also present in French Guiana [41] and Suriname [30]. One of the three infections found in *Ny. whitmani* was characterized as *L.* (*V*.) *guyanensis*. Lainson et al. [33] found five infections of *L. (V.) guyanensis* in *Ny. whitmani* from the Monte Dourado in northern Pará State. These and our results reignite the intriguing hypothesis of Lainson et al. [33] who suggested that *Ny. whitmani* may play a vector role together with *Ny. umbratilis* in the transmission of *L.* (*V*.) *guyanensis*. Moreover, in AP, a single infection from *Ny. umbratilis* captured near Porto Grande was also compatible with *L.* (*V*.) *guyanensis* by morphology and behavior in hamsters [17]. Rangel and Lainson [53] suggested that flagellates found by Lainson et al. [33] in *Ny. whitmani* could have been *L.* (*V*.) *shawi.* Our characterizations by three different methods (IIF-McAb, isoenzyme electrophoresis, and PCR-RFLP) of the isolates from the three available *Ny. whitmani* collected in 1981, that Rangel and Lainson [53] referred to, were all confirmed to be *L.* (*V.*) *guyanensis.* From this, we conclude that it is very unlikely that *L.* (*V*.) *shawi* occurs in the Guiana Shield, as indicated in [55] and that *Ny. whitmani* in fact contributes to *L.* (*V*.) *guyanensis* transmission.

There is strong evidence that *Ny. whitmani sensu lato* is genetically complex [27], with regional differences in the behavior of distinct geographical populations [7]. Its status as a species complex is presently being discussed and it is quite possible that the Guiana Shield population represents a distinct genetic group. Recent results demonstrating the lower Amazon’s “leishmanian bridging zone”, where *L.* (*V.*) *shawi shawi* and *L.* (*V*.) *guyanensis* coexist, as well as a *L.* (*V*.) *guyanensis/L.* (*V.*) *shawi shawi* hybrids [28], support the idea of a genetically different *Ny. whitmani* population in the Guiana Shield.

Natural infections of *Ps. paraensis* found in other regions of the Amazon support its role as a potential vector of *L*. *(V.) naiffi* [2, 34]. However, the absence of such infections in the 104 *Ps. paraensis* in the present work does not negate the presence of *L.* (*V.*) *naiffi* in Serra do Navio.

The finding of *L.* (*V*.) *guyanensis* in a specimen of *Bi. flaviscutellata* from French Guiana [16] raises speculations of potential changes in transmission in the Guiana Shield. The main reason for this is that the classical vectors are arboreal and *Bi. flaviscutellata* is a ground-loving species. One hypothesis is that there are *L.* (*V*.) *guyanensis* infections in both arboreal and terrestrial mammals. *L*. (*L.*) *amazonensis* has been found in this phlebotomine as well as in small mammals and humans in the Guiana Shield [9, 55, 68]. Absence of infections in this species in the present study could be misleading and explained by the fact that only 35 females were dissected. The small numbers in catches are perhaps related to this fly being less attracted to light traps [37]. However, in other situations, CDC sets installed at ground level have collected significant numbers of *Bi. flaviscutellata* [14, 20].

The present rate of 1.43% of *Leishmania*-like infections in *Ny. umbratilis,* with 13 infections proven to be *L.* (*V.*) *guyanensis*, is in accordance with other surveys conducted in French Guiana with 1.3% [40] and Amazons State with 1.04% [51], but higher frequencies were reported in the former territory (15%) [39], as well as in a nearby area on the outskirts of Porto Grande (26%) [17]. These facts do not merely consolidate the role of this species as the main ACL vector in AP, but make us aware of a factual risk of Serra do Navio being a hotspot for ACL transmission. Reported ACL cases have been identified early as being caused by this parasite in the region [23]. In addition, two strains isolated from ACL patients (MHOM/BR/1996/M15781; MHOM/BR/1996/M15937), likely infected in Serra do Navio, with diagnosis performed by our team at the time of field expeditions, were typed by the IIF-McAb technique as *L.* (*V.*) *guyanensis* and are identical to our *Ny. umbratilis* isolates (unpublished observations). The feasible classical mechanism of diurnal infection documented involves females resting on tree bases, and when humans come close to these ecotopes, they may disturb the insects that start aggressive biting behaviors [50].

Single *L.* (*V*.) *guyanensis* infection in *Ny. anduzei* is consistent with its apparently secondary participation in the transmission cycle of Guianan ACL. However, the first finding of this fly harboring *L.* (*V.*) *naiffi* raises the interesting hypothesis of an increasing demand for candidates for transmitting this parasite. Another species, *Ps. hirsutus* (Mangabeira, 1942), has also recently been included in the large list of “microscopically based” suspected vectors of *L.* (*V.*) *naiffi* [65], which is already composed of the other psychodopygians *Ps. paraensis, Ps. ayrozai* (Barretto & Coutinho, 1940), and *Ps. squamiventris s.l*., *Ps. davisi* (Root, 1934), and even the lutzomyian member, *Lu. tortura* Young & Rogers, 1984 [2, 22, 23, 29, 36, 62] (Table 4). “Molecular-based” vector speculations were not included in the former list due to their questionable ability to determine true infection, as recently argued by Brazil et al. [6]. The lack of *Leishmania* typing methods in AP means that our conclusions are biased toward the hypothesis that *L.* (*V.*) *guyanensis* is the principal etiological agent of ACL. However, it is reasonable to assume that ACL cases due to *L.* (*V.*) *naiffi* are underreported in AP as it is normally self-healing and there is no routine identification of ACL parasites. Different ecological situations may affect the incidence of this form of ACL, and in Amazonas State, two of eight *L.* (*V.*) *naiffi* cases did not respond to initial treatment [12].

Two individuals of *Ny. pajoti* from CDC ground were found infected with *Leishmania-*like flagellates, which were not successfully cultured. This species harbors an unidentified *Leishmania* species [10, 31, 74]. Its arboreal behavior coincides with those of *Ny. umbratilis, Ny. anduzei,* and *Ny. whitmani*, all of which have been shown to be infected with *L.* (*V*.) *guyanensis.* This leads us to speculate that the two unknown flagellates in *Ny. pajoti* were also *L.* (*V*.) *guyanensis*. These four fly species were all captured in ground level CDCs, increasing their potential role as vectors to humans during the night.

Despite medical evidence highlighting *Mg. migonei* as a vector of *L.* (*V.*) *braziliensis* in north-eastern and south-eastern Brazil [53], there are no signs of its implication in the context of leishmaniasis in the Amazon region. The isolate from this species was compatible with an unknown trypanosomatid which should be better characterized in the future. Moreover, another similar infection in *Mg. migonei* was recently found during an entomological study in Oiapoque, far north of AP, and although parasite isolation failed, DNA was obtained from the slide used for dissection. In both situations, the PCR-RFLP technique was negative, confirming that these parasites are distinct from *Leishmania* (TV Santos, M Ginouves, G Prévot, personal observation).

Failure of flagellate isolation occurred with *Sc. sordellii* and *Lu. gomezi*. Regarding *Sc. sordellii*, this is not of concern for the ecology of ACL because infection of this species is historically recognized as being caused by other non-*Leishmania* trypanosomatids [56], and even further PCR-based findings of *Leishmania* DNA within this fly [24, 38] did not suggest a determinant role in the transmission of leishmaniasis. In the case of *Lu. gomezi*, however, its epidemiological relevance as a vector of *L.* (*V.*) *panamensis* outside the Brazilian Amazon [13, 66, 71] and PCR-based suspicion of it carrying *L*. (*V*.) *naiffi* in Panama [3] raise the hypothesis that it may be competent in harboring *Leishmania* species from the Guiana Shield. This speculation is supported by its early infection by unidentified flagellate in French Guiana [9], as well as by our findings of a single specimen that proved to be infected with *L.* (*V*.) *shawi shawi* in the lower Amazon region [65].

Trapping methods favor different species, resulting in a bias that portrays the enzootic and/or the zoonotic cycle. In the present study, CDC light trap catches were the highest, but they were set for a greater number of hours. When the captures were corrected for the number per/hour (Table 2), the numbers of *Ny. umbratilis*, *Ps. s. maripaensis*, and *Ny. anduzei* captured in the Shannon trap exceeded those of the CDCs. Even though we were unable to identify all the flagellates found in these three species, we consider that our present and past results add weight to their importance as ACL vectors*.*

The present results provide an update on the phlebotomine fauna inventory of AP and indicate putative ACL vectors for the region. *L.* (*V.*) *guyanensis* infections in *Ny. umbratilis* and *Ny. anduzei* confirmed them, respectively, as primary and secondary vectors. Our analysis of passed infections of *Ny. whitmani* suggests that this species may also participate in the transmission scenario. On the other hand, the circulation of *L.* (*V*.) *naiffi* in *Ps. s. maripaensis*, which is highly anthropophilic, raises the possibility of the occurrence of underreported ACL cases related to this parasite. The finding of *L.* (*V*.) *naiffi* in *Ny. anduzei* adds yet another vector to the long list (Table 4) of suspected vectors of this parasite. The absence of infections in *Bi. flaviscutellata* and *Th. ubiquitalis* does not exclude their possible involvement in ACL transmission in AP. Both are well-known vectors in a nearby region, being associated, respectively, with *L.* (*L*.) *amazonensis* and *L.* (*V.*) *lainsoni*.

Clinical data suggest that the highest risk of infection in AP is from the *L.* (*V.*) *guyanensis* enzootic via a mosaic of vectors, and from the *L.* (*V.*) *lainsoni* enzootics. However, the presence of species considered as putative or proven vectors indicates that there is also a risk of infection from the *L.* (*V.*) *braziliensis, L.* (*V.*) *naiffi,* and *L.* (*L.*) *amazonensis* enzootic cycles, and that more than one vector may be involved in each cycle. It remains to be seen whether the unidentified infections represent infections of known *Leishmania* species in other phlebotomines or infections of new parasite species.

## Conflict of interest

The authors declare that they have no conflict of interest.
